# Piezo-Potential Generation in Capacitive Flexible Sensors Based on GaN Horizontal Wires

**DOI:** 10.3390/nano8060426

**Published:** 2018-06-12

**Authors:** Amine El Kacimi, Emmanuelle Pauliac-Vaujour, Olivier Delléa, Joël Eymery

**Affiliations:** 1 University Grenoble Alpes, CEA, LETI, MINATEC Campus, F-38054 Grenoble, France; amine.elkacimi@gmail.com (A.E.K.); emmanuelle.pauliac-vaujour@cea.fr (E.P.-V.); 2 University Grenoble Alpes, CEA, LITEN, MINATEC Campus, F-38054 Grenoble, France; olivier.dellea@cea.fr; 3 University Grenoble Alpes, CEA, INAC-MEM, Nanostructures and Synchrotron Radiation Laboratory, F-38000 Grenoble, France

**Keywords:** piezoelectricity, sensor, capacitive, wires, GaN, metal-organic vapour phase epitaxy, finite element modelling

## Abstract

We report an example of the realization of a flexible capacitive piezoelectric sensor based on the assembly of horizontal $\bar{c}$-polar long Gallium nitride (GaN) wires grown by metal organic vapour phase epitaxy (MOVPE) with the Boostream^®^ technique spreading wires on a moving liquid before their transfer on large areas. The measured signal (<0.6 V) obtained by a punctual compression/release of the device shows a large variability attributed to the dimensions of the wires and their in-plane orientations. The cause of this variability and the general operating mechanisms of this flexible capacitive device are explained by finite element modelling simulations. This method allows considering the full device composed of a metal/dielectric/wires/dielectric/metal stacking. We first clarify the mechanisms involved in the piezo-potential generation by mapping the charge and piezo-potential in a single wire and studying the time-dependent evolution of this phenomenon. GaN wires have equivalent dipoles that generate a tension between metallic electrodes only when they have a non-zero in-plane projection. This is obtained in practice by the conical shape occurring spontaneously during the MOVPE growth. The optimal aspect ratio in terms of length and conicity (for the usual MOVPE wire diameter) is determined for a bending mechanical loading. It is suggested to use 60–120 µm long wires (i.e., growth time less than 1 h). To study further the role of these dipoles, we consider model systems with in-plane 1D and 2D regular arrays of horizontal wires. It is shown that a strong electrostatic coupling and screening occur between neighbouring horizontal wires depending on polarity and shape. This effect, highlighted here only from calculations, should be taken into account to improve device performance.

## 1. Introduction

Many domains such as electronics [1], optoelectronics [2,3,4] and sensors [5,6,7] are now using semiconductor nanowires, pillars and rods as basic materials for application purposes. In particular, zinc oxide (ZnO) and more recently Gallium nitride (GaN) wires have been demonstrated as efficient building blocks of piezoelectric sensors and energy harvesters [8,9,10,11,12] delivering up to 1.5 V output voltage and 100 nA output current for ZnO [13]. These results may position them as promising technologies to replace or supplement batteries in portable electronics and wireless devices [14,15]. Vertical ZnO wires grown on silicon by the hydrothermal method or solid-vapour process [16,17] have been integrated into rigid and flexible capacitive structures for piezoelectric nano-generators [18,19], and vertical GaN nanowires grown by molecular beam epitaxy (MBE) have been also used to obtain piezo-generators operating under compressive strain [20,21,22]. With these two growth techniques, the wire length is generally quite short due to their intrinsic low growth rates, typically reaching a maximum of 10 µm for hydrothermal methods and less than 5 µm in MBE. In general, this limitation must be overcome to enhance performance and scalability, and sometimes simply to have enough quantity of materials. Recently, it has been shown that it can be achieved with the metal organic vapour phase epitaxy (MOVPE) method that allows reaching several hundred-micron long GaN wires with large aspect ratio (>100) [23]. They can be incorporated in devices both in the vertical geometry with as-grown wires and in the horizontal geometry where the wires are dispersed or oriented on a substrate. To take advantage of vertical devices of the epitaxial relationship, we demonstrated very good performances of capacitive flexible sensor [24] with $\bar{c}$-axis ultra-long GaN vertical wires grown on sapphire and peeled in polydimethylsiloxane before being included in the device. However, it has been also shown that flexible capacitive sensors can also incorporate assembled horizontal wires using both the chemical functionalization in liquids [25] and the Langmuir–Blodgett method to get a monolayer of partially aligned GaN wires [26]. Previously, a straightforward dispersion method (liquid spreading) has been used to orientate ZnO nanowires randomly in the horizontal plane [27]. This previous work has explained some basic principles of the potential generation for the devices working under bending deformation: the output signal was attributed to the summation of the charge separation inside the wires driven by the piezoelectric effect and by the conical shape of the wires. However, up to now, no detailed explanation of the key role of this shape on charge generation has been completely given at the single wire level, as well as the influence of electrostatic interactions occurring between wires and resulting from the distributions of orientations. 

This paper will address the basic understanding of the physical mechanisms involved in capacitive sensors based on horizontal wire-assembly taking an example of the realization of an experimental device and also studying the problem with finite element modelling (FEM). The horizontal assembly ultra-long GaN MOVPE-wires will be performed with the Boostream^®^ assembly technique [28] that provides a solution for mass-production on large surfaces (several square meters). Then, the GaN wire layer transferred with this method will be packed in a capacitive structure to fabricate flexible capacitive sensors. The voltage generation of these sensors will be studied by a surface compression loading/release of several positions on stripe-shaped devices. This solicitation shows a variability that will be tentatively explained in the paper. For this purpose, the general piezoelectricity generation of the wires and its dependence on their local orientation will be studied by using the piezoelectric module of the COMSOL Multiphysics^®^ Simulation Software [10,26]. The importance of the conical shape in potential generation will be clarified and the evolution of the generated potential as a function of wire geometrical parameters (length and conical angle) will be studied for a given mechanical bending. The wire geometry for device efficiency optimization will be discussed to provide a guide for the wires to be grown by MOVPE. The role of the horizontal relative orientation of the wires with respect to their horizontal polarity axis (already addressed for vertical GaN wires [24,29,30] will be studied in detail and several model-configurations of in-plane orientations of the $\bar{c}$-axis wires will be compared. This will be achieved by investigating the electrostatic coupling between wires, when assembled side-by-side. These results will clarify the experimental requirements to get high-performance devices with the assembly of horizontal GaN wires.

## 2. Experiments: Piezoelectricity Measurements on Horizontal Capacitive GaN Wires Devices

**Figure 1:**
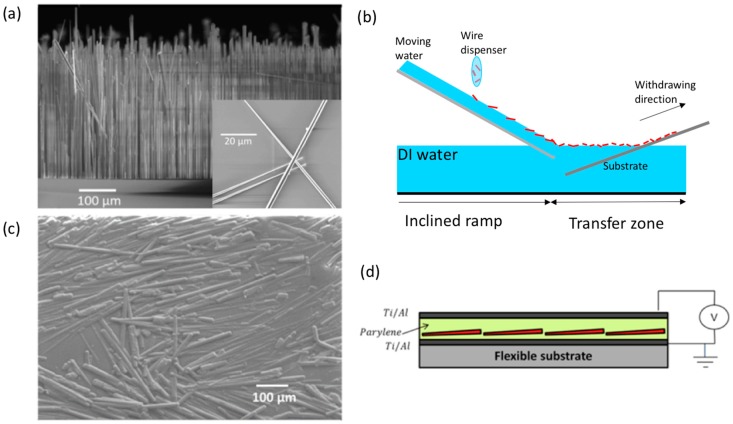
(**a**) Scanning electron microscopy (SEM) image of metal organic vapour phase epitaxy (MOVPE) grown ultra-long wires (~300 µm) grown on sapphire substrate [23]. (**b**) Schematics of the Boostream© process used for the wire assembly. (**c**) SEM image of about 300 µm long GaN wire encapsulated by a thin layer of Parylene-C after Boostream^®^ assembly. (**d**) Schematics for the capacitive device structure using horizontally assembled GaN (in red) and parylene-C dielectric.

GaN wires are grown at CEA on c-sapphire substrate using an AIXTRON showerhead MOVPE reactor with silane addition [31]. This method provides very high growth rates (larger than 100 µm/h) and achieves ultra-long wires with tunable length (controlled from growth duration) and excellent crystalline quality: the threading dislocations created at the substrate interface are bent by the sidewall surface and can be observed only at the bottom of the wires. The details about growth and characterization of the GaN wires grown with this technique are given in References [23,24,26]. They are mainly N-polar with a growth axis is along the GaN $\bar{c}$-direction and have a non-regular hexagonal cross-section with m-plane facet sidewalls (i.e., $\left[10\bar{1}0\right]$ crystallographic planes). Their shape is slightly conical (<2°) and their length can vary from 10 to 700 µm depending on growth time and gas mixture conditions. Figure 1a shows a scanning electron microscopy (SEM) image of ~300 µm long GaN wires used for device fabrication (the growth time is about 3 h). These wires are detached from the sapphire substrate by sonication in an acoustic bath and undergo a chemical functionalization following the procedure reported in Reference [26]. The assembly is achieved using the Boostream^®^ technique that consists of spreading the chemical solution containing wires on a moving liquid (deionized water) flowing down a ramp. Wires trapped at the liquid interface are driven by hydrodynamic forces to the “transfer zone” where they accumulate gradually leading to their self-assembly as a compact monolayer film. The film obtained, thanks to the packing of wires, can be then transferred onto a substrate positioned at the end of the transfer area by applying a slow withdrawing movement (few cm/min) [28].

As depicted on Figure 1b, wires are then accumulated on the transfer zone and transferred to the surface of a pre-processed metallized flexible substrate by withdrawing them vertically in contact with the edge of the accumulation zone. This allows the realization of stripes of wires, whose width is defined by the quantity of wire provided to the dispenser. The substrate is previously coated with a 2 µm thick parylene-C layer [26]. The wire transfer is facilitated by the capillary forces occurring at the edge of the transfer zone in the interface with the substrate. Once the assembly is finished, a second layer of parylene-C is deposited in order to encapsulate the piezoelectric wires into this dielectric (see Figure 1c showing a SEM image of this intermediate state). To create the complete capacitive structure schematized in Figure 1d, a Ti (10 nm)/Al (200 nm) top electrode is finally deposited by physical vapour deposition.

**Figure 2:**
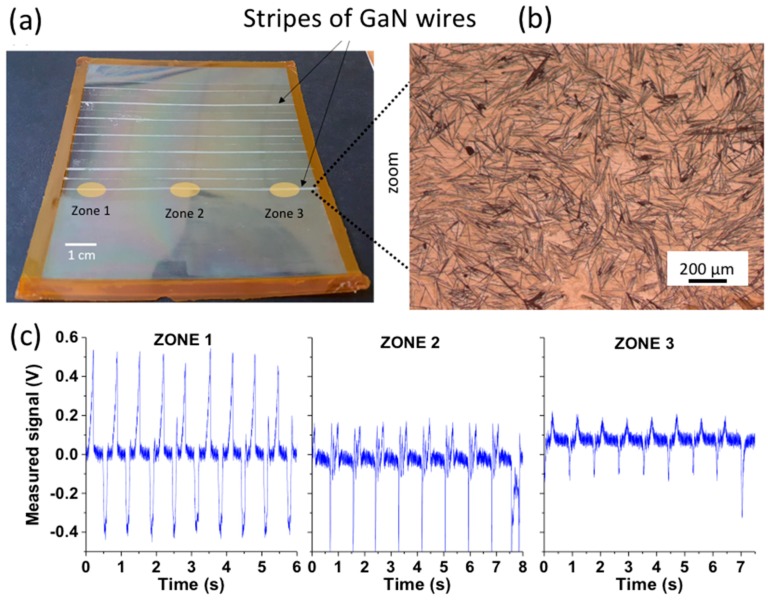
(**a**) Stripes of GaN wires assembled with the Boostream^®^ process. (**b**) Optical microscopy image of the wires. (**c**) Piezoelectric signal measured on three different regions of a flexible devices made of 104 µm long wires (see Figure 1d). The sensor is subjected to a cycled local compression load/release of 1 N/cm² on 1 cm-diameter disk at the speed of 900 mm/min.

A device made of 104 µm long wire (measured by SEM after dispersion) is characterized using an automated mechanical bench (MultiTest 25-I compression setup of the Mecmesin company equipped with a loadcell). The capacitive device structure with 4 µm of total dielectric thickness and metallic contacts (see Figure 1d) is mounted on a 125 µm thick PET substrate. A cycling compression load/release of 1 N/cm² (at a speed of 900 mm/min.) is vertically applied on disks of 1 cm of diameter of a sensor stripe (6 mm width and 11 cm long). Three zones indicated in Figure 2a are measured and their output signals are compared in order to evaluate the degree of variability within the same stripe. 

In these types of measurements, shown here as an illustration, we observe a strong variation in the shape and amplitude of the measured signal as a function of the area of the sensor being compressed. The average peak output signal measured at one end of the device is about 0.6 V while its value is only 0.2 V in the second end. In the middle, we observe a strongly asymmetric signal. This is supposed to be caused by the non-uniform wire assembly along the active area of the device. The average relative orientation and density of the wires might differ from one zone to another due to the transfer at the surface of the liquid and this variability may influence directly the overall output signal delivered by each region. This reveals an intrinsic effect of the process, and more generally this example illustrates several questions that have to be studied in detail:

- What is the physical nature of the piezoelectric signal measured in these capacitive flexible devices based on horizontal GaN wires? What is the role of the size and shape of the wires and the role of the bending amplitude?
- What is the role of the wire density, orientation and interaction?
- What are the best wire features in terms of geometry and configuration to be used in the flexible capacity devices?

In the following, an extensive use of finite element modelling will provide some answers to these questions, by first considering single GaN wires and then interacting wires buried in the complete device stacking. 

## 3. Finite Element Modelling of Piezo-Potential Generation in Single GaN Wires

### 3.1. Method: Geometry and Physical Parameters

**Figure 3:**
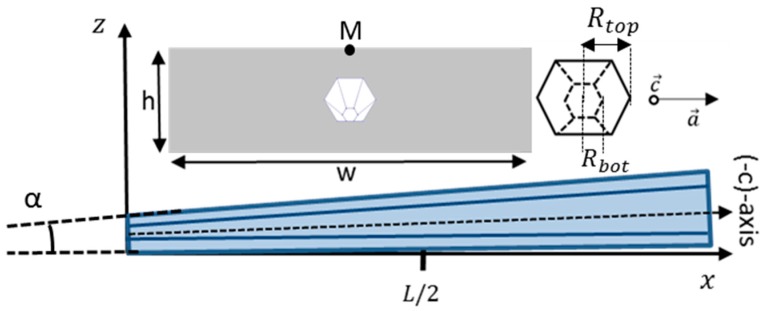
Schematics of the wire geometry (length *L* and conicity angle α) and definition of axes. The left inset gives a front view of the simulated structure consisting of a single conical wire embedded into a dielectric layer of height *h* and with *w*. The right inset is a front view of the conical wire showing its top and bottom diameter $R_{top}$ and $R_{bot}$.

As shown in Figure 3, we will consider cone-shaped wires with a regular hexagonal cross-section with $\left[10\bar{1}0\right]$ crystallographic sidewall planes (called m-plane facets in the literature), which are usually obtained in MOVPE growth. A wire is defined by its length L, conical angle α and top diameter $R_{top}$**.** It is embedded into a Parylene-C dielectric layer and, for the sake of simplification, the thickness h of the dielectric layer is fixed to 2 µm and its width w to 10 µm. As shown in Figure 3, the growth direction of the wire (occurring along the $\bar{c}$-direction for MOVPE wires) forms an angle α with the x-axis. The a-axis of the GaN hexagonal wurtzite structure is oriented along the y-axis. Note that this geometry is closer to crystalline growth than a modelling of the wires with a cylindrical cross-section (as studied in Ref. [27]) and that we do not consider wires grown along the a-axis as discussed in ref. [20]. Their origins are centred at the base of the wire bottom c-plane facet. The conicity angle α is the angle formed between the top sidewall facet of the wire and the bottom horizontal plane having both m-plane crystallographic orientations as shown in Figure 3 (local m-planes kinks are not considered in this study).

Given the usual sizes of the GaN wires obtained by MOVPE growth (diameter larger than 100 nm), we do not expect giant piezoelectric effects predicted in nanostructures [32,33,34] and standard piezoelectric matrix and mechanical elasticity tensor of bulk GaN can be used: $e_{31}=-0.338\;C.m^{-2},\;e_{33}=0.667\;C.m^{-2},\;e_{15}=-0.167\;C.m^{-2}\;\mathrm{and}\;c_{11}=390\;\mathrm{GPa},\;c_{12}=145\;\mathrm{GPa},\;c_{13}=106\;\mathrm{GPa},\;c_{33}=398\;\mathrm{GPa},\;c_{44}=105\;\mathrm{GPa}$ [35,36]. Note that only the piezoelectric polarization is taken into account as the spontaneous polarization of GaN is supposed to be equilibrated by free charges [35,36]. According to experimental data, the young modulus, Poisson ratio, density and relative permittivity of Parylene-C are taken to be 3.2 GPa, 0.4, $1.289\;g.{\mathrm{cm}}^{-3}$ and 3.12, respectively.

### 3.2. Method: Bending Deformation and Potential Generation

A bending deformation, simulating flexible device loading, is applied at the bottom face of the stacking. The displacement profile along the z-axis is described by a second order polynomial $z\left(x\right)=\frac{1}{2*\rho }{\left(x-\frac{L}{2}\right)}^{2}$ leading to a parabolic profile centred on L/2 with a curvature radius ρ. The dielectric/wire/dielectric stacking is bonded on a thick substrate and the resulting neutral line (depending on the thickness of the substrate) is below the dielectric layer. This stacking can be considered to be therefore completely in compression or in tension. For the sake of simplicity, this neutral line is located at the bottom of the dielectric layer in the simulations.

**Figure 4:**
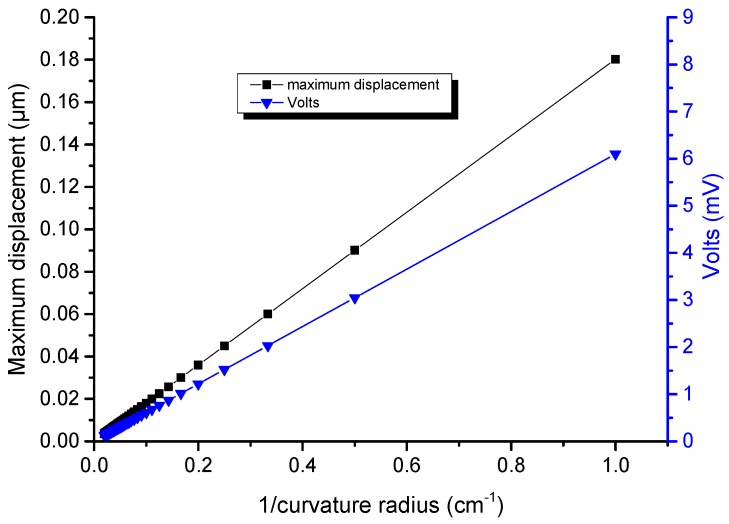
Maximum displacement and output voltage as function of the curvature radius ρ for a single wire embedded in a capacitive structure. The maximum displacement occurs at the wire extremities and the potential value is taken at the middle of the top facet of the wire (see point M in Figure 3).

Figure 4 shows the finite element calculation of the output voltage (*V*) calculated at the top centre of the dielectric (see point M in Figure 3) and of the maximum displacement of a wire ($R_{bot}=400\;\mathrm{nm},\;L=120\;µm$ and α=1°) for a bending curvature radius ρ varying between 1 and 50 cm. *V* varies linearly as a function of 1/ρ as expected from linear elasticity theory. Indeed, for this deformation range, second order piezoelectric effects can be neglected (because the maximum displacement of 180 nm, which occurs at the wire extremities, is smaller than the minimum dimension of the structure, i.e., $R_{bot}$). In the following, the curvature radius will be fixed to ρ=10 cm to discuss the influence of other effects. 

**Figure 5:**
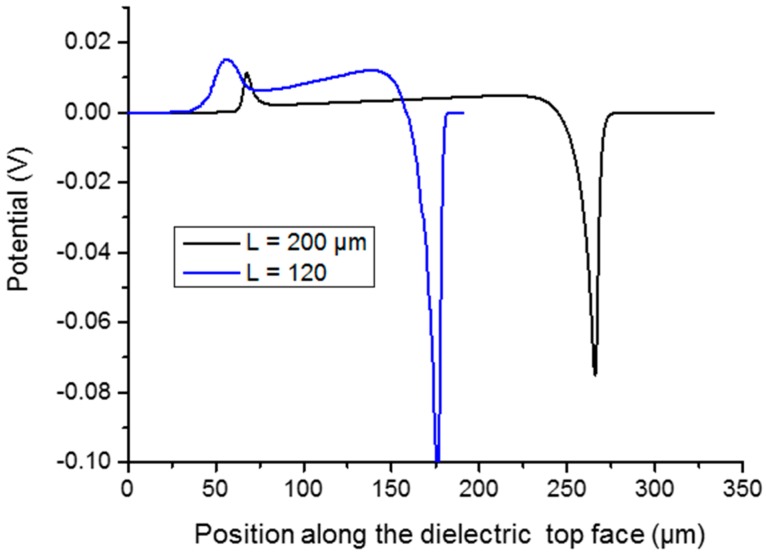
Calculated potential along the top dielectric facet with *L* = 120 and 200 µm long wires embedded in *h* = 2 µm dielectric layer with α = 1° conicity angle and ρ= 10 cm curvature radius bending.

The potential profiles taken at the top of the dielectric layer for single wires with L=120 and 200 µm lengths are reported in Figure 5. They exhibit a “plateau” along most of the wire length and two peaks of opposite signs at the wire extremities resulting from piezoelectric charge accumulations on ±c-plane facets. The plateau’s level and slope vary as the function of L. Such preliminary calculation motivates detailed analysis of the impact of the length L and conical angle α on the wire piezoelectric response. 

### 3.3. Potential Generation Dependence as a Function of Wire Length at Fixed Conicity

The piezoelectric response of a single wire embedded in a 2 µm-thick dielectric layer (see inset of Figure 3) is studied as a function of the wire length *L*. We still consider a wire with hexagonal cross-section having a fixed conicity angle α=1° and a $\mathrm{top}\;\mathrm{diameter}\;R_{top}$. Due to the number of free parameters, the volume and surface of the wire cannot be both fixed together when the length is varied and that makes the comparison of the resulting generated potential difficult. The conditions to get small variations of the values of the wire volume and surface can be fulfilled by adjusting at each step of the parametric study the value of the top diameter $R_{top}$ according to the value of L. In this geometry, the wire volume and surface (neglecting top and bottom c-plane facets) are expressed as follows:

(1) $$\begin{array}{cc} V_{nw}= & \sqrt{3}/2\;L\;R_{top}{}^{2}\;\left(1+f+f^{2}\right),\;S_{nw}=6\;R_{top}L\;\left(1+f\right)/[2\cos\left(\alpha /2\right)]\\ & with\;f=R_{bot}/R_{top} \end{array}$$

**Figure 6:**
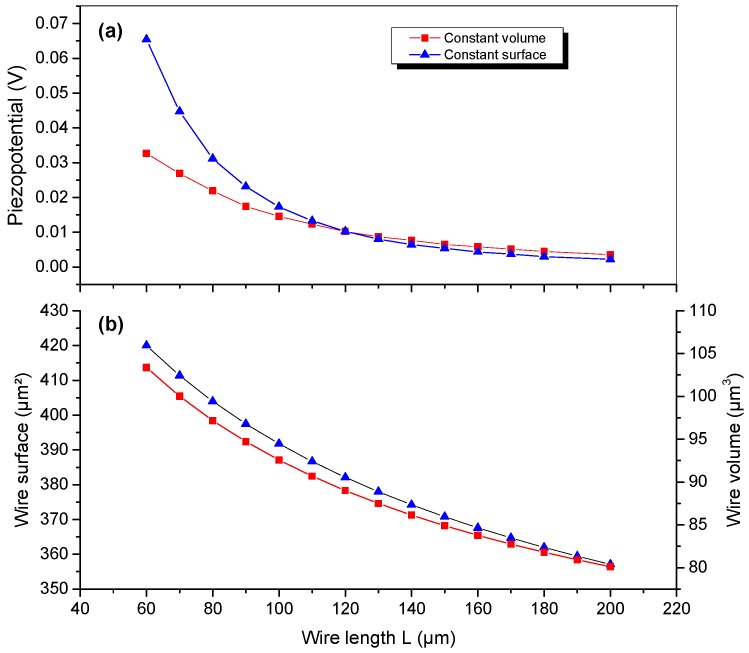
(**a**) Piezo-potential calculated at center of the wire as a function of the wire length for α = 1° and ρ= 10 cm curvature radius; (**b**) the related variation of the wire surface and volume.

To be consistent with usual MOVPE growth morphologies [23,24,26], the origin of the sizes used as a reference for this parametric study is chosen to be $L_{ref}=120\;µm\;\mathrm{and}\;R_{top_{ref}}=700\;\mathrm{nm}$. It corresponds to the volume $V_{ref}=88\;{µm}^{3}$ and the surface $S_{ref}=386\;µm²$. Noting that the (1+f+f²) and (1+f) terms vary between 1.6–2.1 and 1.4–1.6 for L ranging from 50 to 200 µm, we can deduct from Equation (1) that $L\;R_{top}{}^{2}$ and *L* *R_top_* can be assumed constant. Consequently, the conditions of constant volume and constant surface can be roughly approximated by changing the top wire diameter according to $R_{top}=R_{top_{ref}}\;\sqrt{L_{ref}/L}\;\mathrm{and}\;R_{top}=R_{top_{ref}}\;L_{ref}/L$. For these two approximations, we plot in Figure 6a the generated piezo-potential measured at the middle of the wire as a function of the wire length L for a fixed conicity angle (α=1°). A monotonous decrease of the piezo-potential as a function of L is obtained for both constant volume and constant surface approximations (note that the crossing of the two curve occurs of course at the point corresponding to the reference). The largest discrepancy is obtained for the smaller wire length, but these approximations are roughly equivalent beyond 120 µm. These calculations indicate that shorter lengths (in the 60–120 µm range) should be preferred for device fabrication.

As shown in Figure 6b, the assumptions chosen to demonstrate the intrinsic effect of length (in the 60–200 µm range) on the potential level are justified *a posteriori* by the variations of the wire volume (surface), which are limited to 20% (16%).

### 3.4. Potential Generation Dependence as a Function of Wire Conicity at Fixed Length

**Figure 7:**
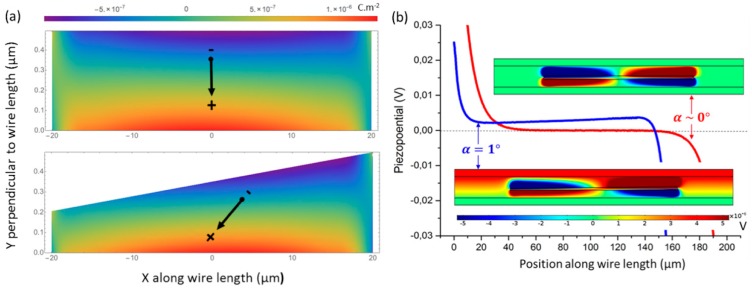
Two-dimensional finite element calculation of non-conical (α = 0°) and conical (α = 1°) embedded GaN wires bended under 10 cm radius curvature. (**a**) Piezoelectric charge density mappings (note the different scales along X and Y). (**b**) Piezo-potential taken at the top m-plane facet al.ong the length for (lines) and the corresponding cross-section mappings of the potential across the structure in insets.

The role of a conical shape in generating potential in horizontal wire capacitive geometry was already pointed out in the literature [27]. The disorientation of the $\bar{c}$-axis wire direction with respect to the bottom surface was claimed to favour charge generation, but a detailed analysis of this effect has not yet been provided. To study this point, we map in Figure 7 the electrostatic charge and the piezo-potential inside conical (α=1°) and a non-conical (α=0°) GaN wires with L=120 µm and $R_{top}=700\;\mathrm{nm}$ embedded in a 2 µm thick Parylene-C layer for a bending deformation (ρ=10 cm).

Calculations are performed with a 2D model without a loss of generality with the displacement profile given in Section 3.2. According to Figure 7a, the GaN wire exhibits a top (bottom) region with negative (positive) charge densities that can be integrated at the first order to get an equivalent dipole (represented by an arrow). The symmetry of the charge density distribution with respect to the middle of the wire observed for a non-conical shape is broken for the conical configuration. As shown in the insets of Figure 7b, the charge density asymmetry has a large influence on the piezo-potential generation: without the conical shape, the piezo-potential is fully symmetric with respect to the centre of the wire and no difference of potential appears between the top and bottom of the dielectric layers. This effect explains the zero-slope profile along the wires drawn in Figure 7. When this symmetry is broken by a slight conical shape, positive and negative charges regions do not fully compensate anymore and a potential drop occurs between electrodes. The sign of this difference of potential depends on the bending direction. As seen in Figure 7b, the piezo-potential plateau value is different from zero with a slope along the wire length for α ≠ 0. Of course, these simplified calculations are performed in an ideal case. In real devices, wires will have different curvatures and an origin taken in the middle of the wire may not be fulfilled; nevertheless, all these local effects will contribute to determine the equipotential of metallic electrodes. Note also that this mechanism is a little bit more complicated than what it is involved in the vertical wire-based device, which can operate both in compression mode (the deformation along the vertical axis is directly induced by the applied force) and in the bending mode, where the strain along the principal axis of the wire is only imposed by the Poisson’s effect [24].

**Figure 8:**
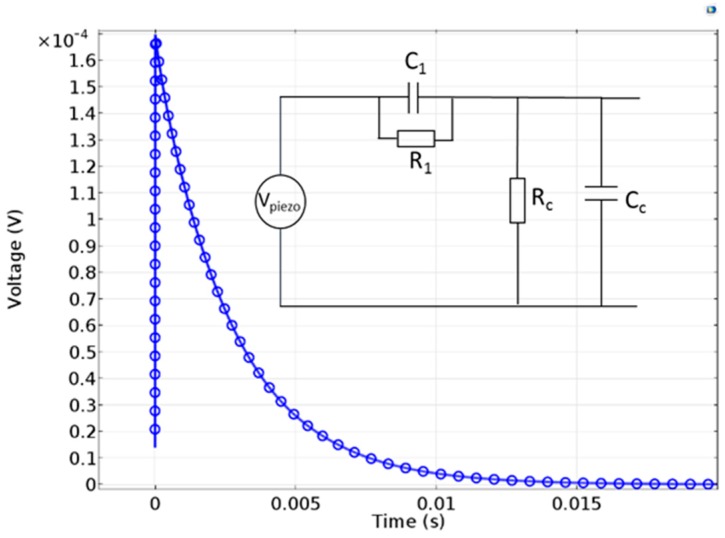
Time-dependent behaviour of the wire-based sensor obtained by finite element modelling simulation for a structure with a single cone-shaped wire of 120 µm length and 1° conicity angle under 10 cm curvature radius bending. The voltage is taken on the top electrode while the bottom is grounded. The inset shows the equivalent electrical circuit of the device. (*R*_1_, *C*_1_) correspond to the internal impedance of the sensor and (*R*_C_, *C*_C_) to the measurement setup.

In agreement with the present experimental measurements and Reference [26], when a fast bending is applied to the wire, a charge splitting is created during the deformation with a fast-transient time imposed by electrostatics. The sign of the resulting potential depends on the concave or convex curvature. When this deformation is maintained, this distribution can reach its zero-charge equilibrium in a slower process due to charge redistribution. Such behaviour can be modelled though an equivalent electrical model (with *R*_1_ = 70 MΩ and *C*_1_ = 200 pF) and also by finite element calculations as shown in Figure 8.

Due to the number of free parameters, it is again not possible to study the effect of the conical angle at a given length (L=120 µm) without changing considerably the volume (53% in the 0.1–2° α-range) and the surface (62%) of the wires. To minimize the choice of the constant surface or volume assumptions, we searched a criterion to limit their ratio variation. By neglecting the surface of the top and bottom facets of the wire, the total wire surface corresponds to six times the surface of a single lateral polygonal facet $S_{nw}=6\;\left[L\;Rtop\;\left(1+f\right)\right]/\left[2\;cos\left(\alpha /2\right)\right]$. With a first order approximation in α, the $S_{nw}/V_{nw}$ ratio can be approximated by

(2) $$\frac{S_{nw}}{V_{nw}}=\frac{6}{\sqrt{3}}\frac{\left(1+f\right)}{R_{top}\left(1+f+f^{2}\right)}$$

**Figure 9:**
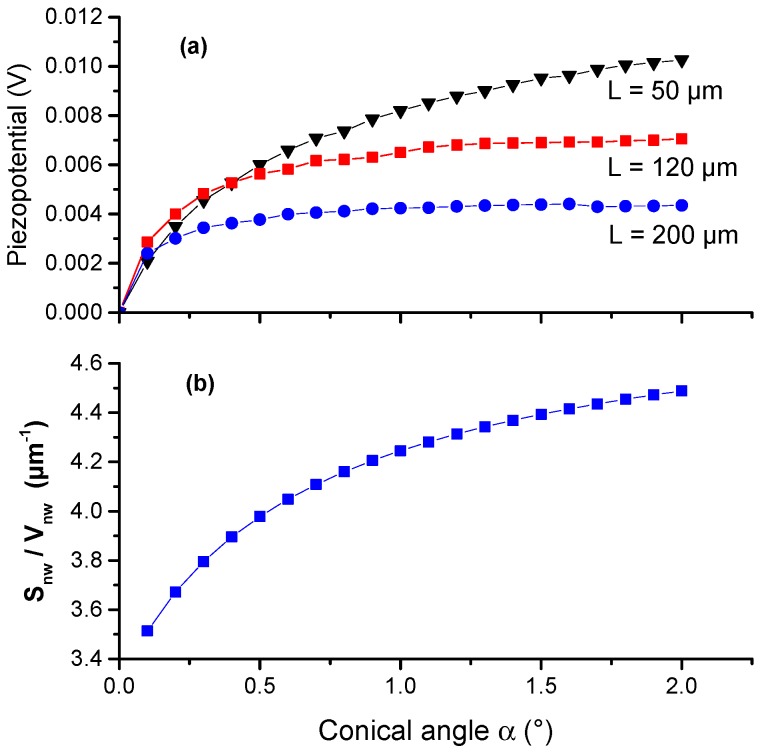
(**a**) Piezo-potential evolution as function of the conicity angle α for wires with *L* = 50, 120, 200 µm and and $R_{top}=700\;\mathrm{nm}$. (**b**) The related variation of the surface over volume ratio.

The factor (1+f)/(1+f+f²) can be neglected in Equation (2) because its variation is limited to 25% in the 0.1–2° α-range as shown in Figure 7b and the ratio $S_{nw}/V_{nw}$ can be roughly approximated by $6/\left(\sqrt{3}\;R_{top}\right)$, i.e., by keeping the top diameter constant. The evolution of the piezo-potential measured at the top dielectric facet (see schematics in Figure 3) as a function of α is shown in Figure 9 for three different wires lengths L=50, 120, 200 µm for $R_{top}=700\;\mathrm{nm}$.

Whatever the length, we observe a monotonous increase of the potential as a function of α followed by a saturation at larger values. The relative variation is stronger for angles ranging between 0.1 and 0.5° and for shorter wire length (see L=50 µm). As a result, the saturation is reached at smaller values of α for longer wires. It occurs around 0.8° for L=200 µm and around 1° for L=120 µm, while we still observe significant potential increase beyond 1.5° for L=50 µm. From these calculations, we can conclude that a quite small value of α is sufficient to break the symmetry of the charge distributions and then to induce the charge collection. A large conical angle (α>1.2°) does not improve considerably the voltage output for long wires. Practically, quite short wire (for example *L* < 100 µm), i.e., with 1–1.5° conical angle must be preferred for device efficiency optimization. These target values are doubtlessly achievable in MOVPE because of spontaneous anti-tapering shape obtained during growth [23,24,26].

## 4. Finite-Element Modelling of the Complete Capacitive Stacking with Metallic Contacts and Wire Assemblies

As illustrated in Figure 1c, a large number of wires are usually assembled together within the dielectric layers [25,26]. Therefore, the output voltage of the device strongly depends on the electrostatic coupling and charge screening occurring between wires, which are directly governed by the wire density (i.e., inter-distance) and the relative orientation with respect to their polarity [26,27,29]. Several assembly techniques have been proposed to control the relative orientation and density of the wires, while keeping growth polarities [25]. However, these techniques generally result in broad orientation distributions with variable density and size [27]. In the following, we will study by finite element calculations the physics of the electrostatic coupling between wires in the horizontal assembly to quantify the influence of different wire orientations on the generated potential.

### 4.1. Method: Simulated Structure and Calculation of Periodic Boundary Conditions

**Figure 10:**
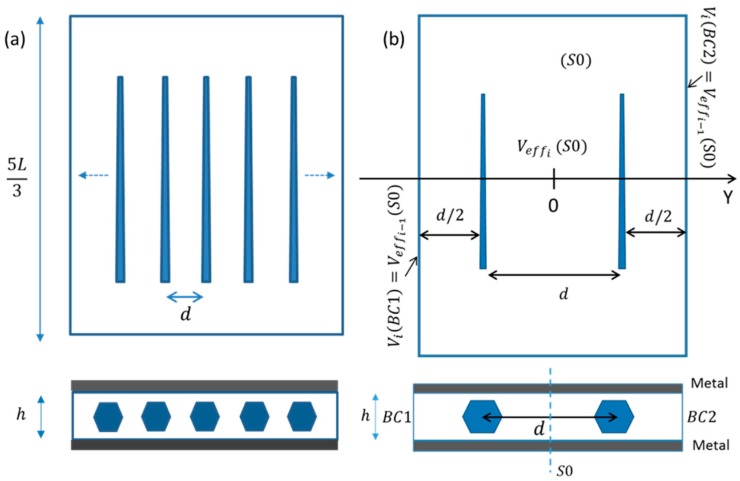
(**a**) Schematics of a regular horizontal assembly of wires. (**b**) Elementary cell structure composed of two wires illustrating the boundary condition calculation method.

Let us consider an ideal assembly of horizontal GaN wires with the same orientation lying in the X-Y plane, periodically arranged along the Y-axis at a constant distance d from each other and separated by 2L/3 along the X-axis. As shown in Figure 10a, this assembly is buried within 2 µm thick Parylene-C based capacitive structure with top and bottom metal electrodes. Free boundary conditions are applied on X-axis. 

**Figure 11:**
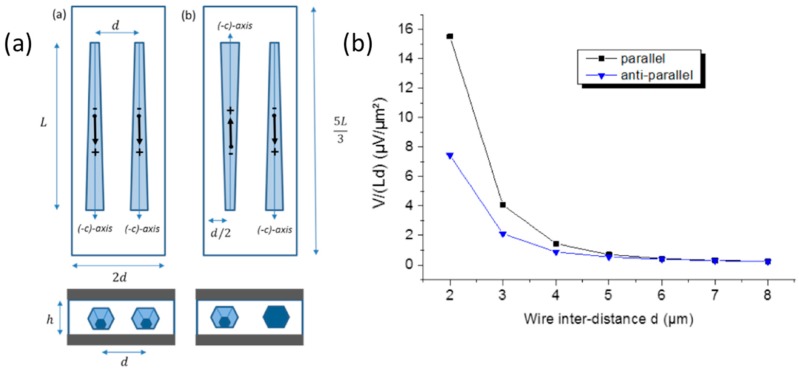
(**a**) Schematics of the wire relative crystallographic orientation: (**a**) parallel and (**b**) anti-parallel configurations of the $\bar{c}$ growth axis. The m-planes of GaN are parallel to the bottom electrode. The black arrows in the center of the wires are the in-plane representations of the equivalent electric dipoles schematized in Figure 7. (**b**) Potential per surface area measured between metal electrodes as function of wire inter-distance d for parallel and anti-parallel configuration of the wire orientation. A bending deformation of 10 cm curvature radius is applied to the bottom part of the simulated structure.

Due to symmetry consideration, the simulation can be performed with a self-consistent iterative process on a two-wire elementary cell by implementing periodic boundary conditions on the lateral faces of the structure. The choice of two wires instead of only one enables us to consider hereafter both parallel and anti-parallel orientations of the $\bar{c}$-growth axis of the wires, as shown in Figure 11. The self-consistent iterative process schematized in Figure 10b consists of the calculation of the average potential value V(S0) between the wires at the surface S0 and re-injecting this value as a boundary condition (V(BC1) and V(BC2)) in the next iterative step. This process is repeated n-times until a converging value of the boundary conditions is obtained. Once convergence is reached (n is generally lower than 20), electrostatic periodicity condition is fulfilled and we have V(S0)=V(BC1)=V(BC2). The actual potential can then be extracted from the top metal electrode.

### 4.2. Screening of Electric Fields in a One-Dimensional Network

**Figure 12:**
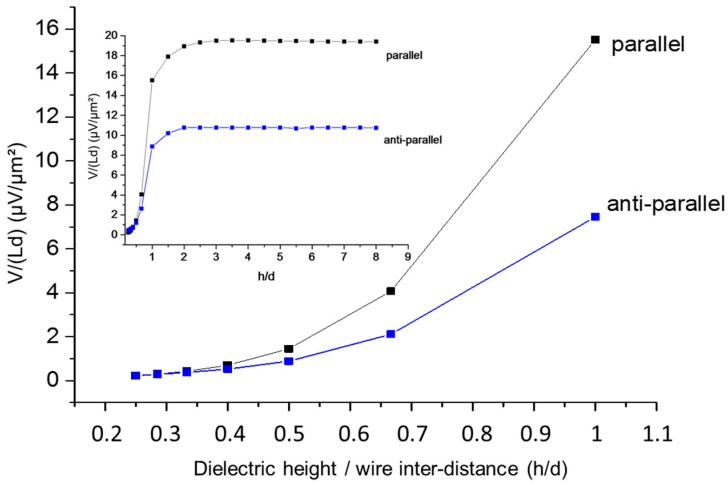
Surface potential as function of the parameter *h/d* for a two-wires in parallel and anti-parallel configurations under a bending with ρ = 10 cm curvature radius. The potential is taken at the top electrode while the bottom one is grounded (see Figure 8). The graph focuses on values of *h/d* lower than 1. The inset graph shows a zoom out for the whole range of values of *h/d* from 0.25 to 8.

Based on this method, the potential generated in parallel and anti-parallel orientations is calculated as a function of the wire inter-distance d for the geometries described in Figure 11a (the term parallel corresponds to a similar orientation of the $\bar{c}$-axis vectors of the wires). The dielectric layer is 2d large, 5L/3 long and of a thickness h. The evolution of the generated potential V per surface unit as function of d for these two configurations is shown in Figure 12. The variable *d* varies between 2 and 8 µm whereas fixed values are chosen for L=120 µm, $R_{top}=700\;\mathrm{nm},$ α=1° and h=2 µm. 

It is demonstrated in both configurations that a small value of the wire inter-distance d (for example d<5 µm) corresponding to a high wire surface density is preferable to get a high potential. In addition, the parallel configuration provides a larger output at a small *d.* Indeed, for d=2 µm, the generated potential between metal electrodes is V=4.5 mV for the parallel configuration and only 2 mV for the anti-parallel one. We also notice that the two configurations provide the same output level for values of the inter-distance beyond a value of 5 µm corresponding to lower density. For such interspacing, we can suppose that wires are not interacting anymore because of a low density and consequently this calculation provides an estimation of the interaction length of the electrostatic field in this geometry. The electrostatic screening between the wires is governed by the inter-distance d, but also by the wires’ position with respect to the electrode, defined by the dielectric layer height h (see schematics in Figure 10). The variation of the generated potential V per surface area is drawn in Figure 12 as a function of h/d for both parallel and anti-parallel configurations with a special focus on the 0.2–1 range, which corresponds to thin and dense wire devices (the inset al.so shows higher values of h/d).

In agreement with previous results, this figure gives evidence that the parallel configuration is more efficient. The generated potential becomes negligible when the thickness of the capacitive structure h is roughly smaller than half of the distance separating the wires, while higher values are reached when the device thickness tends to be of the same order than the wire inter-distance d. This illustrative calculation shows how much attention must be paid to the device design and that the dielectric height must be adjusted in device fabrication with respect to the densities achieved during assembly. 

### 4.3. Screening of Piezoelectric Fields in a Two-Dimensional Network

**Figure 13:**
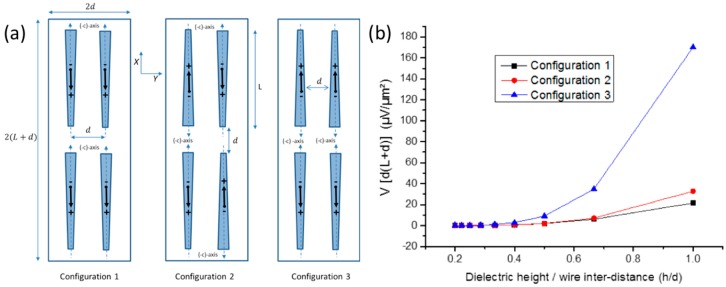
(**a**) Schematics of three in-plane arrays of two-dimensional wire assemblies. The black arrows sketch the equivalent electric dipoles shown in Figure 7 and Figure 11. (**b**) Comparison of the evolution of the normalized potential by unit surface as function of *h/d* for configurations shown in (**a**). The value of *h* is fixed at 2 µm while d is varied between 2 and 10 µm.

To introduce a second level for the description of the assembly, we now consider an elementary cell of the device consisting of four wires separated by an inter-distance d, in X and Y directions, integrated into capacitive structure. The elementary cell is 2(L+d) long, 2d wide and the inter-distance d is varied. The three different configurations schematized in Figure 13a are considered to mimic the experimental layouts. As the elementary cell is part of a two-dimensional network of wires, boundary conditions in terms of potential are applied on the four edges (along X- and Y-directions). These boundary conditions were calculated using the iterative method described previously. In the first configuration (*C*1), wires are assembled in a parallel orientation along both X- and Y-axes, while in the second configuration (*C*2), wires are anti-parallel along both directions. In *C*3, parallel (anti-parallel) orientation is applied along X-axis (Y-axis).

The potentials generated by these three configurations are compared for the same mechanical bending (ρ=10 cm) as a function of dimensionless parameter h/d in order to estimate the related electrostatic coupling and screening effects. The normalized output voltage V per unit surface is given in Figure 13b as a function of h/d for L=120 µm, $R_{top}=700\;\mathrm{nm}$ and α=1°. The height *h* was fixed at 2 µm while the wire inter-distance d was varied in the 2−10 µm range. The difference of output potential between configurations (C1) and [(C2) & (C3)] shows the importance of the relative wire orientation along the X-axis, along which the bending is applied. Indeed, Figure 13b shows that the change of the relative orientation along X induces a large variation of the potential. The discrepancy increases with h/d corresponding to denser assemblies or larger dielectric thickness. This effect can be attributed to the interactions between equivalent dipoles described in Figure 7 (and schematized in Figure 13). The overall electrostatic energy of an assembly of dipoles depends on their positioning and relative orientation with respect to polarity. Due to the screening effect, electrostatic fields generated by each of the dipoles may add up or cancel out each other’s and the overall potential varies accordingly. Experimentally, configuration 3, which provides the highest output in this 2D geometry, cannot be obtained using usual assembly process but requires very complex methods such as electrophoresis and micromanipulation. Simple model calculations demonstrate here that the assembly process is a key step and that wires polarities should be precisely controlled to improve the efficiency of horizontally assembled wires-based capacitive devices. This point has not been optimized up to now by using Langmuir-Blodgett [26], the Boostream^®^ technique [28] and other assembling techniques [25]. It should be the focus of further experimental studies. 

## 5. Conclusions

The realization of flexible capacitive piezoelectric sensor based on the assembly of horizontal MOVPE $\bar{c}$-polar GaN wires has been demonstrated with the Boostream^®^ technique [28]. A layer of long wires (>100 µm) that can be textured in stripes is embedded into parylene and the voltage is measured between metallic electrodes deposited on both top and bottom sides. The piezoelectric signal (<0.6 V) obtained by a surface compression/release shows a quite large variability along the stripes. We attributed this behaviour to the distribution of the wire dimensions and in-plane orientations and we studied these assumptions by finite element calculations.

In a first step, the operating mechanisms of these flexible capacitive mechanical sensors have been studied with the linear piezoelasticity theory and bulk parameters in the static and time-dependent regime. In the FEM calculations, we took into account the GaN crystalline orientation ($\bar{c}$-axis growth), the faceting (m-plane surfaces) and the hexagonal cross-section shape of the usual wires grown by MOVPE. We underlined the influence of wire geometry on the generated piezo-potential and output level for a bending loading that corresponds to the usual mechanical solicitation of flexible devices. A slight conical shape (α from 0.5 to 1°) is demonstrated to be necessary and sufficient to optimize the output signal. It creates an equivalent dipole with an in-plane component that breaks the symmetry occurring in perfectly hexagonal wires with α=0 and provides a difference of potential between the electrodes. It shows that a larger conicity angle does not improve significantly the device response, especially for long wire (L>120 µm). To optimize piezo-potential generation, wires with lengths ranging between 60 and 100 µm are preferred. This target geometry (in terms of length and conicity) can be reached by the MOVPE technique evidencing a fast growth rate on very large area [23,24,26]. To optimize the MOVPE mass-production of these materials, a balance can be done between growing ultra-long wires and break them during sonication or grow smaller wires during a shorter time (to minimize the growth cost). We demonstrate here that the in-plane relative orientation of $\bar{c}$-axis of the wires has a strong influence on the sensor efficiency. Indeed, the output signal level calculated for 2D wire networks depends on the electrostatic interaction between equivalent dipoles, which orientation is directly related to the polarity (and conicity for MOVPE growth). This gives a clear insight about attention that must be paid to the assembly process in horizontal devices in order to optimize voltage outputs and hence the overall device performance. These physical findings can be straightforwardly generalized to ZnO wires that are also used for device realizations.
